# Prevention of Hospital-Acquired Infections Among Pediatric Patients: A Scoping Review Protocol

**DOI:** 10.3390/children13060794

**Published:** 2026-06-09

**Authors:** Imanul Hassan Abdul Shukor, Nurul Farehah Shahrir, Nur Khairah Badaruddin, Normala Salim, Sri Devi Sukumaran

**Affiliations:** 1 Institute for Health Management, National Institutes of Health, Ministry of Health Malaysia, Shah Alam 40170, Selangor, Malaysia; nurkhairah.b@moh.gov.my (N.K.B.); dr.sridevi@moh.gov.my (S.D.S.); 2 Environmental Health Research Centre, Institute for Medical Research, National Institutes of Health, Ministry of Health Malaysia, Shah Alam 40170, Selangor, Malaysia; farehah.shahrir@moh.gov.my; 3 Medical Development Division, Ministry of Health Malaysia, Putrajaya 62000, Wilayah Persekutuan Putrajaya, Malaysia; normala.salim@moh.gov.my

**Keywords:** patient safety, clinical governance, risk management, nosocomial infection, adolescence, inpatient care, children, healthcare-associated infections

## Abstract

**Highlights:**

**What are the main findings?**

- Outlines a comprehensive protocol to map the existing literature on strategies and interventions for preventing hospital-acquired infections (HAI) in pediatric patients.
- Details the methodology for evaluating current clinical practices, environmental factors, and vulnerable demographics within pediatric infection control.

**What are the implication of the main findings?**

- Provides a standardized, reproducible search strategy and data extraction plan for conducting scoping reviews in pediatric healthcare settings.
- Serves as a foundational reference for scholars aiming to pinpoint existing knowledge deficits and prioritize future research areas in pediatric HAI prevention.

**Abstract:**

**Background/Objectives**: Hospital-Acquired Infections (HAI) represent one of the most frequent adverse events during care delivery, with the pediatric population (0–18 years) presenting unique vulnerabilities due to their developing immune systems, dependence on caregivers, and need for invasive devices. Despite the availability of general guidelines, existing high-level evidence is largely extrapolated from adult studies, and pediatric settings differ significantly in patient physiology and equipment size. This scoping review aims to map the key concepts, types of evidence, and research gaps related to strategies preventing HAI in pediatric patients. **Methods**: This scoping review will be conducted in accordance with the Joanna Briggs Institute (JBI) methodology and the Preferred Reporting Items for Systematic Reviews and Meta-Analyses Extension for Scoping Reviews (PRISMA-ScR) guidelines. The Population, Concept, and Context (PCC) framework will be utilized. We will include any strategy, intervention, or protocol aimed at preventing HAI. A comprehensive search will be conducted across ten major electronic databases and grey literature sources. Two independent reviewers will screen titles, abstracts, and full texts, followed by data extraction using a standardized tool to categorize the interventions and key findings. **Results**: The findings will synthesize diverse practices into a usable format for clinical decision-makers and identify gaps where primary pediatric research is lacking. This consolidated data aims to guide resource allocation and assist hospital infection control committees in updating pediatric safety protocols. **Conclusions**: This scoping review will establish a comprehensive baseline of pediatric-specific HAI prevention strategies. The findings will inform evidence-based practice, identify critical research gaps, and guide future investigations in the prevention of pediatric infections in healthcare settings.

## 1. Introduction

Hospital-acquired infections (HAI), also referred to as healthcare-associated infections or nosocomial infections, are infections not present or incubating at the time of admission that patients acquire during the course of receiving healthcare treatment for other conditions [1]. In pediatric populations, HAI represents a significant threat to patient safety and clinical outcomes, with unique epidemiological and clinical characteristics that distinguish them from adult HAI [2]. The global burden of HAI in pediatric populations is substantial, though precise epidemiology varies significantly by geographic region, healthcare setting, resource availability, and patient population characteristics. In developing countries, HAI rates in pediatric intensive care units are notably elevated compared to high-income nations, reflecting disparities in infection control infrastructure, staffing ratios, and resource availability [3].

The prevention of hospital-acquired infections in pediatric populations has evolved substantially over the past two decades, with increasing recognition that infection control strategies must be tailored to the unique physiological, developmental, and care delivery characteristics of children [2,4]. Current evidence encompasses a range of interventions, from fundamental hand hygiene practices to sophisticated technological devices and comprehensive quality improvement initiatives [5]. Despite these advances, significant challenges persist in translating evidence-based interventions into consistent practice across diverse pediatric settings. Infection control in pediatrics must address unique factors including respiratory virus infections (particularly respiratory syncytial virus and influenza), rotavirus, varicella zoster virus, and pertussis, which represent persistent challenges in children’s hospitals [6]. Additionally, pediatric-specific considerations such as breastmilk handling, toys, therapy animals, and parental involvement in care delivery introduce infection risks not typically encountered in adult settings [6].

Despite substantial progress in pediatric HAI prevention, significant research gaps persist, limiting the evidence base for optimal prevention strategies in children [2,7]. There is insufficient evidence for safe and uniform protocols in several areas of pediatric HAI prevention. A scoping review of oral hygiene practices in pediatric intensive care units (PICU) found inadequate evidence for a safe and uniform oral hygiene protocol in PICU children, with a notable gap regarding oral hygiene in non-ventilated children [8]. Further studies are needed to support the development of uniform, safe, effective, and evidence-based protocols for children in PICU [8]. In addition, research is needed to better understand HAI prevention in specific pediatric subpopulations, including immunocompromised children (oncology, bone marrow transplant), surgical populations, and children with chronic conditions requiring long-term device use [9,10]. Additionally, research on infection prevention and control measures for preterm infants discharged into the community remains limited, with cohesive guidance not clearly established [11]. These research gaps underscore the critical need for systematic reviews and scoping reviews to map existing evidence, identify knowledge deficits, and guide future research priorities in pediatric HAI prevention [12].

While existing syntheses have extensively evaluated infection control within specific subsets, such as NICU-specific protocols [3], PICU safety bundles [4], or target outcomes like CLABSI reduction [13], there is a distinct lack of comprehensive evidence mapping across the broader pediatric landscape. Previous reviews predominantly focus on clinical effectiveness (risk-of-bias and effect sizes) within isolated units [3,7]. The primary aim of this scoping review is to systematically identify, map, and synthesize the existing literature on hospital-acquired infection prevention strategies specifically designed for pediatric patients aged 0–18 years across diverse healthcare settings. This scoping review will address three specific objectives: (1) to comprehensively identify and map all available literature addressing hospital-acquired infection prevention interventions, strategies, and programs specifically targeting pediatric patients from birth through 18 years of age; (2) to systematically categorize and describe the types of interventions employed for HAI prevention in pediatric populations; and (3) to examine how HAI prevention strategies vary across different pediatric clinical settings and patient populations.

## 2. Materials and Methods

This scoping review will be conducted in accordance with the methodological framework established by the Joanna Briggs Institute (JBI) for scoping reviews [14] and will adhere to the Preferred Reporting Items for Systematic Reviews and Meta-Analyses extension for Scoping Reviews (PRISMA-ScR) checklist [15] (Table S1). The JBI framework provides structured guidance on defining the research question, establishing eligibility criteria, conducting comprehensive searches, selecting evidence, extracting data, and presenting results [14]. Prior to commencement of the study, the scoping review protocol was formally registered in the Open Science Framework registry (Identifier: 10.17605/OSF.IO/82CWF).

### 2.1. Eligibility Criteria

Eligibility criteria for this scoping review have been developed using the Population, Concept, and Context (PCC) framework recommended by the JBI for scoping reviews [15].

#### 2.1.1. Population

The population of interest comprises pediatric patients aged 0–18 years who are at risk of or have experienced hospital-acquired infections in any healthcare setting. This broad age range encompasses the full spectrum of pediatric development, including neonates (0–28 days), infants (29 days to <1 year), toddlers (1–2 years), preschool children (3–5 years), school-age children (6–12 years), and adolescents (13–18 years). Studies focusing on any pediatric subpopulation within this age range will be eligible for inclusion, including those targeting specific clinical groups such as critically ill neonates, immunocompromised children, surgical patients, or those with chronic conditions requiring prolonged hospitalization. Studies will be included regardless of the underlying medical condition, severity of illness, or reason for hospitalization, provided that the focus is on HAI prevention in the pediatric population. Studies that include both pediatric and adult populations will be eligible if pediatric-specific data can be extracted. Studies conducted exclusively in adult populations (≥19 years) will be excluded.

#### 2.1.2. Concept

The concept of interest is the prevention of hospital-acquired infections in pediatric patients, which includes, but is not limited to, central line-associated bloodstream infections (CLABSI), catheter-associated urinary tract infections (CAUTI), ventilator-associated pneumonia (VAP), surgical site infections (SSI), and other device-associated or procedure-related infections. Studies will be included if they describe, evaluate, or report on any intervention, strategy, or program aimed at preventing HAI in pediatric populations. Both single-component and multicomponent interventions will be eligible. Studies may employ any research design, including randomized controlled trials, quasi-experimental studies, observational studies (cohort, case–control, cross-sectional), quality improvement reports, implementation studies, and mixed-methods research. Qualitative studies exploring barriers and facilitators to HAI prevention will also be included. Studies will be excluded if they focus solely on the treatment of established infections without addressing prevention, or if they examine infections acquired outside the healthcare setting (e.g., community-acquired infections).

#### 2.1.3. Context

The context for this review encompasses any pediatric healthcare setting where hospital-acquired infections may occur. Studies will be included regardless of geographic location, healthcare system type (public, private, academic, community), or resource setting (high-income, middle-income, or low-income countries). This inclusive approach will enable the review to capture the full diversity of HAI prevention strategies implemented across different healthcare contexts and resource environments.

The time period of January 2000 to present was selected to capture contemporary HAI prevention practices while maintaining a manageable scope. Table 1 shows the inclusion and exclusion criteria.

### 2.2. Data Collection

#### 2.2.1. Electronic Databases

The following electronic bibliographic databases will be searched from January 2000 to May 2026:

- PubMed;
- Cochrane Library;
- Scopus;
- Web of Science Core Collection;
- Cumulative Index to Nursing and Allied Health Literature (CINAHL);
- Embase;
- WHO Institutional Repository for Information Sharing;
- OpenGrey;
- Google Scholar;
- ProQuest Dissertations;
- Other relevant institutional or governmental reports or registries.

#### 2.2.2. Search Strategy

The search strategy will combine three main concept groups using Boolean operators. Table 2 describes in detail the proposed search strategy keywords.

All search strategies will be documented in full in an appendix to ensure transparency and reproducibility. Search results will be exported to Zotero reference management software, and duplicates will be removed using both automated and manual deduplication processes. An example of a proposed search strategy using the PubMed database is presented in Table 3.

### 2.3. Study Selection

Study selection will follow a rigorous two-stage screening process conducted by independent reviewers to minimize bias and ensure consistency in the application of eligibility criteria.

#### 2.3.1. Stage 1: Title and Abstract Screening

Following deduplication, two independent reviewers will screen the titles and abstracts of all retrieved records against the predefined eligibility criteria. Screening will be conducted using Rayyan web platform (www.rayyan.ai, accessed on 12 May 2026), utilizing its semi-automated, machine-learning-based prediction classifier to assist with study prioritization [16], which facilitates independent, blinded screening and automatic conflict resolution workflows. Each record will be classified as “include,” “exclude,” or “maybe” by each reviewer. Records classified as “include” or “maybe” by at least one reviewer will proceed to full-text review. Records classified as “exclude” by both reviewers will be removed from further consideration.

#### 2.3.2. Stage 2: Full-Text Review

All records that pass title and abstract screening will undergo full-text review by two independent reviewers. Full-text articles will be retrieved and assessed against the complete set of eligibility criteria. Reviewers will document the primary reason for exclusion for each excluded study using predefined exclusion categories (e.g., wrong population, wrong concept, wrong context, wrong publication type, language, no full text available). This information will be used to populate the PRISMA-ScR flow diagram and will provide transparency regarding the reasons for study exclusion.

#### 2.3.3. Conflict Resolution

At both screening stages, disagreements between reviewers will be resolved through discussion and consensus. If consensus cannot be reached, a third senior reviewer will be consulted to make a final determination. All conflicts and their resolutions will be documented in Rayyan to maintain an audit trail of decision-making throughout the study selection process.

#### 2.3.4. Documentation and Reporting

**Figure 1:**
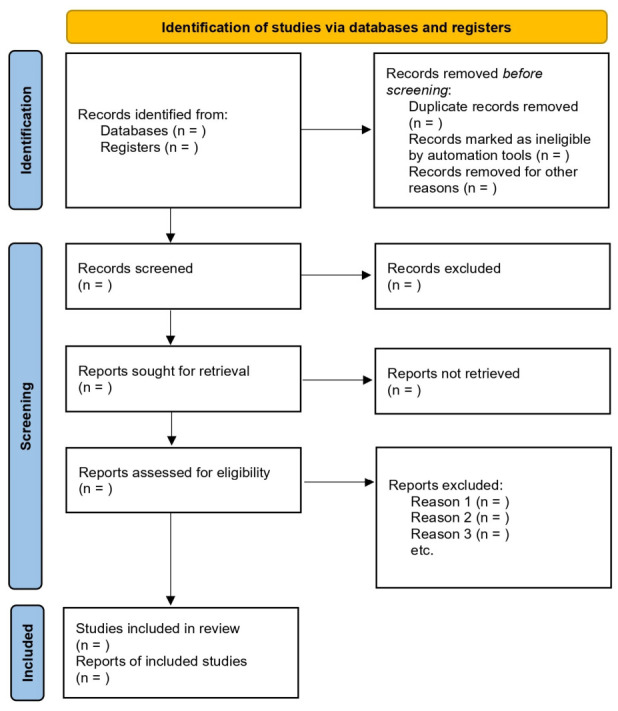
PRISMA flow diagram.

The study selection process will be documented using a PRISMA-ScR flow diagram, which will report the number of records identified, screened, assessed for eligibility, and included in the final review, along with reasons for exclusion at the full-text stage [17]. This transparent reporting will enable readers to understand the scope of the literature search and the rationale for inclusion and exclusion decisions. Figure 1 shows the PRISMA-ScR flow diagram.

### 2.4. Data Extraction

The data charting form will be developed collaboratively by the research team based on the review objectives and the PCC framework. The form will be designed to capture both descriptive characteristics of included studies and substantive findings related to HAI prevention interventions. The data charting process will be iterative, as recommended by JBI guidance for scoping reviews [15]. As reviewers become familiar with the included studies, additional data elements may be identified as relevant and added to the charting form. Any modifications to the form will be documented and applied retrospectively to previously charted studies to ensure consistency. During the data charting phase, extracted interventions will be descriptively grouped using a preliminary clinical framework. This guiding framework will consider categories such as hand hygiene, device-associated infection bundles, antimicrobial stewardship, environmental cleaning, isolation precautions, vaccination, caregiver education, and surveillance systems, while remaining flexible to accommodate emerging or overlapping strategies. Table 4 describes the data elements to be extracted from each included study.

### 2.5. Data Synthesis

Consistent with scoping review methodology, data synthesis will be primarily descriptive and narrative in nature, with the aim of mapping the characteristics and extent of the literature rather than aggregating effect estimates. The synthesis will provide a comprehensive overview of HAI prevention interventions in pediatric populations, identify patterns and trends in the evidence base, and highlight gaps and areas for future research.

A narrative synthesis approach will be used to organize and present the substantive findings of included studies. Studies will be grouped thematically and within each thematic category, a narrative summary will describe the range of interventions implemented, the contexts in which they were applied, the outcomes measured, and the reported effectiveness. Patterns of convergence and divergence in findings will be identified and discussed.

To ensure standardization and comparability, all hospital-acquired infections and device-associated modules were defined strictly according to the NHSN Surveillance Definitions [18]:

1. Hospital-Acquired Infection (HAI): An infection is classified as an HAI if the date of event (onset of symptoms or diagnostic specimen collection) occurs on or after the third calendar day of admission to the facility (with the day of admission being Day 1), and there was no evidence that the infection was present or incubating upon admission.
2. Central Line-Associated Bloodstream Infection (CLABSI): Defined as a laboratory-confirmed bloodstream infection (LCBI) where an eligible central line (or umbilical catheter in neonates) was in place for more than 2 calendar days on the date of the event and was in place on the date of the event or the day before. For pediatric patients below 1 year of age, LCBI criteria include at least one clinical symptom (e.g., fever, hypothermia, apnea, or bradycardia) combined with a matching pathogen from blood cultures or a common skin contaminant isolated from two separate blood cultures.
3. Catheter-Associated Urinary Tract Infection (CAUTI): Defined as a urinary tract infection where an indwelling urinary catheter was in situ for more than 2 calendar days on the date of the event, with a positive urine culture with no more than two species of organisms, accompanied by at least one clinical sign (fever for children; or hypothermia, apnea, bradycardia, dysuria, or suprapubic tenderness for infants less than 1 year of age).
4. Ventilator-Associated Pneumonia (VAP): Defined as a pneumonia developing in a patient mechanically ventilated for more than 48 h, demonstrated by a combination of radiologic criteria (new or progressive infiltrates, consolidation, or cavitation) and clinical or microbiological criteria (temperature instability, leukopenia or leukocytosis, new onset of purulent sputum, or increased suctioning requirements).
5. Surgical Site Infection (SSI): Classified as superficial incisional, deep incisional, or organ/space infection occurring within 30 or 90 days after an operative procedure, characterized by purulent drainage, positive cultures from the site, or explicit diagnosis by the attending surgeon.

To enhance the accessibility and interpretability of findings, results will be presented using a combination of tables, figures, and diagrams:

- PRISMA-ScR flow diagram: Documenting the study selection process from identification through to final inclusion.
- Summary tables: Presenting key characteristics of included studies (author, year, country, design, setting, population, intervention, outcomes, key findings).
- Frequency tables: Showing the distribution of studies by year, country, setting, intervention type, and HAI type.
- Evidence maps: To provide a visual overview of the evidence, heat maps and bubble plots will be generated using Python (version 3.14) within the Google Colab environment, utilizing the Seaborn (version 0.13.2) and Matplotlib packages (version 3.10.0). The code notebook will be made publicly accessible to ensure reproducibility.
- Thematic diagrams: Conceptual frameworks or logic models illustrating relationships between intervention components, implementation strategies, and outcomes.

In alignment with standard scoping review methodology, a formal critical appraisal or risk-of-bias assessment of the included studies will not be conducted, as the primary objective of this study is to map the existing landscape of literature rather than synthesize evidence quality.

## 3. Expected Results and Implications

This scoping review is anticipated to possess several methodological strengths that will enhance the validity, comprehensiveness, and utility of its findings. The broad scope of the review, encompassing all pediatric age groups (0–18 years), all clinical settings, and all intervention types, is expected to provide a comprehensive overview of the pediatric HAI prevention landscape that has not been previously synthesized. Unlike systematic reviews that focus on specific interventions or infection types, this scoping review is designed to map the breadth of evidence, identify patterns and gaps, and inform future research priorities [15,19].

The decision to conduct a scoping review rather than a systematic review was informed by several methodological considerations. First, the pediatric HAI prevention literature encompasses a broad spectrum of interventions—ranging from care bundles and educational programs to technological devices and antimicrobial stewardship initiatives—each evaluated across different clinical contexts and patient subpopulations [13,20,21]. Second, the objective of this review is not to synthesize effect sizes or generate pooled estimates of intervention efficacy, but rather to provide a comprehensive overview of what is known, identify gaps in the evidence base, and inform future research priorities [22]. Third, scoping reviews do not mandate formal quality appraisal of included studies, allowing for the inclusion of diverse evidence types (e.g., observational studies, quality improvement reports, implementation studies) that collectively contribute to understanding the landscape of HAI prevention in pediatric care [23,24].

The inclusive approach to clinical settings and age groups is expected to reveal important variations in HAI epidemiology and prevention strategies across the pediatric spectrum. By examining evidence from NICUs, PICUs, general wards, and outpatient settings, and by considering neonates, infants, children, and adolescents separately, the review is positioned to identify age- and setting-specific gaps that may inform targeted research and quality improvement efforts.

## 4. Conclusions

This scoping review protocol outlines a comprehensive and systematic approach to mapping the evidence on hospital-acquired infection prevention in pediatric patients aged 0–18 years. The significance of this scoping review lies in its potential to inform evidence-based practice, guide policy development, and catalyze future research in pediatric HAI prevention. By providing a comprehensive synthesis of what is known and what remains unknown, the review is expected to serve as a foundational resource for infection prevention and control teams, clinicians, researchers, policymakers, and quality improvement leaders working to enhance the safety of pediatric healthcare delivery.

## Figures and Tables

**Table 1:** Inclusion and exclusion criteria.

Criterion	Inclusion	Exclusion
Population	Pediatric patients aged 0–18 years in any healthcare setting; studies with mixed populations if pediatric-specific data extractable	Studies exclusively in adult populations (≥19 years); studies with no extractable pediatric data
Concept	Any intervention, strategy, or program aimed at preventing hospital-acquired infections; all study designs including randomized controlled trials, observational studies, implementation studies, qualitative research	Studies focusing solely on treatment of established infections; studies examining community-acquired infections only; studies without clear HAI prevention focus
Context	Any pediatric healthcare setting; any geographic location or resource setting	Non-healthcare settings (e.g., schools, homes) unless linked to discharge planning or post-hospitalization infection prevention
Publication type	Peer-reviewed journal articles, conference proceedings with full text, secondary studies (e.g., systematic reviews, meta-analyses, scoping reviews), case series, case reports, and grey literature (e.g., dissertations, theses, government reports, preprints)	Editorials, commentaries, opinion pieces, letters to the editor, abstracts without full text, protocols without results
Language	English language publications	Publications without English full-text article
Time period	January 2000 to present (May 2026)	Publications before January 2000

HAI: Hospital-acquired infections.

**Table 2:** Search strategy keywords.

Group	Terms
Pediatric population	Including MeSH terms (Infant, Newborn; Infant; Child, Preschool; Child; Adolescent; Pediatrics) and keywords (neonat*, infant*, child*, paediatric*, pediatric*, adolescent*, newborn*, baby, babies, toddler*)
Hospital-acquired infection	Including MeSH terms (Cross Infection; Healthcare-Associated Infections; Catheter-Related Infections; Surgical Wound Infection; Pneumonia, Ventilator-Associated) and keywords (hospital-acquired infection*, healthcare-associated infection*, nosocomial infection*, HAI, HCAI, CLABSI, CAUTI, VAP, SSI, device-associated infection*)
Prevention and intervention	Including MeSH terms (Primary Prevention; Infection Control; Quality Improvement; Patient Care Bundles) and keywords (prevent*, intervention*, bundle*, protocol*, checklist*, stewardship, hand hygiene, quality improvement, care bundle*)

MeSH: Medical Subject Headings; HAI: Hospital-acquired infections; HCAI: Healthcare associated infections; CLABSI: Central line-associated bloodstream infections; CAUTI: Catheter-associated urinary tract infections; VAP: Ventilator associated pneumonia; SSI: Surgical site infections. * Asterisks (*) indicate truncation/wildcard symbols used in the search strategy to capture various word endings.

**Table 3:** Proposed search strategy for PubMed database.

Group	Search String
Pediatric population (#1)	(“Infant, Newborn”[Mesh] OR “Infant”[Mesh] OR “Child, Preschool”[Mesh] OR “Child”[Mesh] OR “Adolescent”[Mesh] OR “Pediatrics”[Mesh] OR neonat*[tiab] OR infant*[tiab] OR child*[tiab] OR paediatric*[tiab] OR pediatric*[tiab] OR adolescent*[tiab] OR newborn*[tiab] OR baby[tiab] OR babies[tiab] OR toddler*[tiab])
Hospital-acquired infection (#2)	(“Cross Infection”[Mesh] OR “Healthcare-Associated Infections”[Mesh] OR “Catheter-Related Infections”[Mesh] OR “Surgical Wound Infection”[Mesh] OR “Pneumonia, Ventilator-Associated”[Mesh] OR “hospital-acquired infection*”[tiab] OR “healthcare-associated infection*”[tiab] OR “nosocomial infection*”[tiab] OR HAI[tiab] OR HCAI[tiab] OR CLABSI[tiab] OR CAUTI[tiab] OR VAP[tiab] OR SSI[tiab] OR “device-associated infection*”[tiab])
Prevention and intervention (#3)	(“Primary Prevention”[Mesh] OR “Infection Control”[Mesh] OR “Quality Improvement”[Mesh] OR “Patient Care Bundles”[Mesh] OR prevent*[tiab] OR intervention*[tiab] OR bundle*[tiab] OR protocol*[tiab] OR checklist*[tiab] OR stewardship[tiab] OR “hand hygiene”[tiab] OR “quality improvement”[tiab] OR “care bundle*”[tiab])
Final combined search string with year and language filter	#1 AND #2 AND #3 AND English[Language] AND (“2000”[Date—Publication]: “3000”[Date—Publication])

* Asterisks (*) indicate truncation/wildcard symbols used in the search strategy to capture various word endings. # The hash symbol (#) followed by a number denotes the specific line number from the database search history.

**Table 4:** Data extraction from included studies.

Type of Variable	Data Elements
Study characteristics	Author(s) and year of publication; country and geographic region; country income-level; healthcare resource setting; study design and methodology; study setting; study duration and data collection period; funding source and conflicts of interest
Population characteristics	Age range and developmental stage; sample size; clinical characteristics; underlying conditions or diagnoses; inclusion and exclusion criteria
Intervention characteristics	Type of HAI prevention intervention; specific components of multicomponent interventions; theoretical or conceptual framework guiding the intervention; implementation strategies and processes; duration and intensity of the intervention; healthcare professionals involved in delivery; barriers and facilitators to implementation
Outcome measures	Types of HAI assessed; outcome measurement methods and definitions; baseline and post-intervention HAI rates; statistical significance of findings; effect sizes; secondary outcomes; process measures
Key findings	Summary of main results; authors’ conclusions regarding intervention effectiveness; reported limitations; implications for practice and research

Note: Hospital-acquired infections (HAI).

## Data Availability

No new data were created or analyzed in this study. Data sharing is not applicable to this article.

## Supplementary Materials

The following supporting information can be downloaded at https://www.mdpi.com/article/10.3390/children13060794/s1. Table S1: Preferred Reporting Items for Systematic reviews and Meta-Analyses extension for Scoping Reviews (PRISMA-ScR) Checklist [15].

## Abbreviations

The following abbreviations are used in this manuscript:

HAI	Hospital-acquired infections
PCC	Population, Concept and Context
NICU	Neonatal Intensive Care Unit
PICU	Pediatric Intensive Care Unit
JBI	Joanna Briggs Institute
CLABSI	Central Line-Associated Bloodstream Infections
CAUTI	Catheter-Associated Urinary Tract Infections
VAP	Ventilator-Associated Pneumonia
SSI	Surgical Site Infections
MeSH	Medical Subject Headings
PRISMA-ScR	Preferred Reporting Items for Systematic Reviews and Meta-Analyses Extension for Scoping Reviews

## References

[1] Siegel J.D., Rhinehart E., Jackson M., Chiarello L., Health Care Infection Control Practices Advisory Committee (2007). 2007 Guideline for Isolation Precautions: Preventing Transmission of Infectious Agents in Health Care Settings. Am. J. Infect. Control.

[2] Siegel J.D., Grossman L., Long S.S. (2008). Chapter 2—Pediatric Infection Prevention and Control. Principles and Practice of Pediatric Infectious Disease.

[3] Rosenthal V.D., Dueñas L., Sobreyra-Oropeza M., Ammar K., Navoa-Ng J.A., de Casares A.C.B., de Jesús Machuca L., Ben-Jaballah N., Hamdi A., Villanueva V.D. (2013). Findings of the International Nosocomial Infection Control Consortium (INICC), Part III: Effectiveness of a Multidimensional Infection Control Approach to Reduce Central Line-Associated Bloodstream Infections in the Neonatal Intensive Care Units of 4 Developing Countries. Infect. Control Hosp. Epidemiol..

[4] Wagh A., Sinha A. (2018). Prevention of Healthcare-Associated Infections in Paediatric Intensive Care Unit. Childs Nerv. Syst..

[5] Elward A.M., McGann K.A. (2002). Steps to Reduce Nosocomial Infections in Children. Infect. Med..

[6] Posfay-Barbe K.M., Zerr D.M., Pittet D. (2008). Infection Control in Paediatrics. Lancet Infect. Dis..

[7] Lona-Reyes J.C., Cruz-Chávez T.A., Gallegos-Marín J.A., Chávez-Vázquez A.M., Alatorre-Rendón F., González-Carmona J., Moreno-Medina B. (2025). Healthcare-related infections in a pediatric intensive care unit in Mexico: Epidemiology and associated factors. Rev. Argent. Microbiol..

[8] Alves F.B.T., Pomini M.C., da Silva Santos P.S., Chicrala G.M., Júnior L.A.V.S. (2024). Oral Hygiene Practices in Pediatric Intensive Care Units: A Scoping Review. Rev. Enferm. UFPI.

[9] Bibart M., Eisel E.A., Taylor K., Olshefski R., Camacho C., Welty M., Gajarski R., Guinipero T. (2025). Beyond the Bundle: Reducing Central Line–Associated Bloodstream Infections on a Pediatric Hematology, Oncology, and Bone Marrow Transplant Unit. J. Pediatr. Hematol. Oncol. Nurs..

[10] Kamity R., Grella M., Kim M.L., Akerman M., Quintos-Alagheband M.L. (2021). From Kamishibai Card to Key Card: A Family-Targeted Quality Improvement Initiative to Reduce Paediatric Central Line-Associated Bloodstream Infections. BMJ Qual. Saf..

[11] Carruthers K., Hannis D., Robinson J., Armstrong A. (2023). Infection Prevention and Control Measures for Preterm Infants Discharged into the Community: A Scoping Review Protocol. Syst. Rev..

[12] Molina García A., Cross J.H., Fitchett E.J.A., Kawaza K., Okomo U., Spotswood N.E., Chiume M., Ezeaka V.C., Irimu G., Salim N. (2022). Infection Prevention and Care Bundles Addressing Health Care-Associated Infections in Neonatal Care in Low-Middle Income Countries: A Scoping Review. eClinicalMedicine.

[13] Pageler N.M., Longhurst C.A., Wood M., Cornfield D.N., Suermondt J., Sharek P.J., Franzon D. (2014). Use of Electronic Medical Record–Enhanced Checklist and Electronic Dashboard to Decrease CLABSIs. Pediatrics.

[14] Peters M.D.J., Marnie C., Tricco A.C., Pollock D., Munn Z., Alexander L., McInerney P., Godfrey C.M., Khalil H. (2020). Updated Methodological Guidance for the Conduct of Scoping Reviews. JBI Evid. Synth..

[15] Tricco A.C., Lillie E., Zarin W., O’Brien K.K., Colquhoun H., Levac D., Moher D., Peters M.D.J., Horsley T., Weeks L. (2018). PRISMA Extension for Scoping Reviews (PRISMA-ScR): Checklist and Explanation. Ann. Intern. Med..

[16] Ouzzani M., Hammady H., Fedorowicz Z., Elmagarmid A. (2016). Rayyan—A Web and Mobile App for Systematic Reviews. Syst. Rev..

[17] Page M.J., McKenzie J.E., Bossuyt P.M., Boutron I., Hoffmann T.C., Mulrow C.D., Shamseer L., Tetzlaff J.M., Akl E.A., Brennan S.E. (2021). The PRISMA 2020 Statement: An Updated Guideline for Reporting Systematic Reviews. BMJ.

[18] Centers for Disease Control and Prevention (2026). National Healthcare Safety Network (NHSN) Patient Safety Component Manual.

[19] Peters M.D.J., Godfrey C.M., Khalil H., McInerney P., Parker D., Soares C.B. (2015). Guidance for Conducting Systematic Scoping Reviews. Int. J. Evid. Based Healthc..

[20] Rosenthal V.D., Ramachandran B., Villamil-Gómez W., Armas-Ruiz A., Navoa-Ng J.A., Matta-Cortés L., Pawar M., Nevzat-Yalcin A., Rodríguez-Ferrer M., Yıldızdaş R.D. (2012). Impact of a Multidimensional Infection Control Strategy on Central Line-Associated Bloodstream Infection Rates in Pediatric Intensive Care Units of Five Developing Countries: Findings of the International Nosocomial Infection Control Consortium (INICC). Infection.

[21] Miller M.R., Niedner M.F., Huskins W.C., Colantuoni E., Yenokyan G., Moss M., Rice T.B., Ridling D., Campbell D., Brilli R.J. (2011). Reducing PICU Central Line–Associated Bloodstream Infections: 3-Year Results. Pediatrics.

[22] Munn Z., Peters M.D.J., Stern C., Tufanaru C., McArthur A., Aromataris E. (2018). Systematic Review or Scoping Review? Guidance for Authors When Choosing between a Systematic or Scoping Review Approach. BMC Med. Res. Methodol..

[23] Pollock D., Hasanoff S., Barker T.H., Clyne B., Tricco A.C., Booth A., Godfrey C., Khalil H., Jia R.M., Taneri P.-E. (2025). Over 1000 Terms Have Been Used to Describe Evidence Synthesis: A Scoping Review. BMJ Evid. Based Med..

[24] Silva A., e Silva V.S., Padilha M.I., Petry S., Mendes K.D.S., Costa I.C.P. (2024). Mapping Knowledge in Scoping Reviews: A Guide for Nursing Researchers and Academics. Texto Contexto Enferm..

